# Self-Assembling Nanovaccine Enhances Protective Efficacy Against CSFV in Pigs

**DOI:** 10.3389/fimmu.2021.689187

**Published:** 2021-07-21

**Authors:** Ze-Hui Liu, Hui-Ling Xu, Guang-Wei Han, Li-Na Tao, Ying Lu, Su-Ya Zheng, Wei-Huan Fang, Fang He

**Affiliations:** ^1^ Institute of Preventive Veterinary Sciences & College of Animal Sciences, Zhejiang University, Hangzhou, China; ^2^ Department of Veterinary Medicine, Zhejiang Provincial Key Laboratory of Preventive Veterinary Medicine, Zhejiang University, Hangzhou, China

**Keywords:** infectious diseases, nanoparticle-based technology, nanovaccine, enhanced immunogenicity, protective efficacy

## Abstract

Classical swine fever virus (CSFV) is a highly contagious pathogen, which pose continuous threat to the swine industry. Though most attenuated vaccines are effective, they fail to serologically distinguish between infected and vaccinated animals, hindering CSFV eradication. Beneficially, nanoparticles (NPs)-based vaccines resemble natural viruses in size and antigen structure, and offer an alternative tool to circumvent these limitations. Using self-assembling NPs as multimerization platforms provides a safe and immunogenic tool against infectious diseases. This study presented a novel strategy to display CSFV E2 glycoprotein on the surface of genetically engineered self-assembling NPs. Eukaryotic E2-fused protein (SP-E2-mi3) could self-assemble into uniform NPs as indicated in transmission electron microscope (TEM) and dynamic light scattering (DLS). SP-E2-mi3 NPs showed high stability at room temperature. This NP-based immunization resulted in enhanced antigen uptake and up-regulated production of immunostimulatory cytokines in antigen presenting cells (APCs). Moreover, the protective efficacy of SP-E2-mi3 NPs was evaluated in pigs. SP-E2-mi3 NPs significantly improved both humoral and cellular immunity, especially as indicated by the elevated CSFV-specific IFN-γ cellular immunity and >10-fold neutralizing antibodies as compared to monomeric E2. These observations were consistent to *in vivo* protection against CSFV lethal virus challenge in prime-boost immunization schedule. Further results revealed single dose of 10 μg of SP-E2-mi3 NPs provided considerable clinical protection against lethal virus challenge. In conclusion, these findings demonstrated that this NP-based technology has potential to enhance the potency of subunit vaccine, paving ways for nanovaccine development.

## Introduction

Classical swine fever (CSF), characterized by typical clinical symptoms including fever, anorexia, ataxia and respiratory problems, can result in high morbidity and mortality in pigs (1, 2). Outbreaks of CSF led to significant economic losses in the pig industry worldwide, including Central and South America, Africa and Asia. Consequently, CSF is economically important and listed as a notifiable disease by the World Organization of Animal Health (OIE). Emerging studies have shown that genotype 2 CSFV has replaced genotype 1 and has become the dominant genotype in China (3, 4). Persistent infections caused by chronic and atypical in field conditions are refractory to vaccination *via* the mechanism known as superinfection exclusion (SIE), thus making the epidemic situation of CSFV more complicated (5).

Lapinized attenuated C-strain is considered safe and effective in eliciting cellular and humoral immune response (6). Specifically, IFN-γ induced by C-strain closely contributes to the protection against CSFV infection in the early stage (7). However, C-strain vaccine does not allow serological differentiation between infected and vaccinated animals (DIVA), which severely hinder the control and eradication of CSFV (6, 8). CSFV E2 protein is located on the surface of the viral envelope and involved in the viral infection process. As the main protective antigen of CSFV, E2-based subunit vaccine can induce protective neutralizing antibodies, and it allows DIVA by monitoring anti-CSFV E^rns^ antibodies (9). Hence, it is the preferred target for the development of subunit vaccines (10, 11).

Traditional inactivated or attenuated live vaccines are efficacious in stimulating the immune responses, though usually with biosecurity risks accompanied, such as reversion to virulence and recombination with field strains. Subunit vaccines of simple composition could not cause adverse effects like traditional whole pathogen based vaccines, which facilitates the application in clinical usage (12, 13). Due to the relatively weak immunogenicity, vaccine formulations containing only subunit proteins may not be completely efficacious for humans and other large animals. Innovative nanotechnology has gained attention in the field of vaccine development, which is considered one of the most effective strategies to improve the low immunogenicity of epitope-based vaccines (14, 15).

Self-assembly of viral capsid/envelope proteins into NPs had been proposed for the generation of Virus-like particles (VLPs). As they are non-infectious and non-replicating with an ideal diameter of 20–100 nm, they have been applied in diagnosis and target delivery for drug, DNA, peptide and vaccine. The potential of VLPs in vaccine development has been well demonstrated in the commercialization of human papillomavirus (HPV), hepatitis B virus and malaria vaccines (16). Besides virus derived assemblies, live organisms produce a range of proteins which are able to self-assemble *in situ* into nano-structures with specific biological functions. Following the self-assembly concept, a series of self-assembling proteins has been proposed for vaccine and biomedical applications, including ferritin (17, 18), vault (19, 20), flagellin (21), encapsulin (22) and lumazine synthase (23).

Nanovaccines are new classes of vaccines that have been developed by conjugation of antigens onto NPs *via* a series of strategies, such as chemical modification and genetic fusion. Chemical coupling faces challenges from the heterogeneity in coupling site and physicochemical properties of particles (24, 25). Chemical coupling efficiency varies with the size of the display antigen. Genetic modifications are considered powerful tools in manipulating the outer surface of VLPs and can elicit defined downstream responses (26, 27). Using NPs as delivery scaffolds, multiple copies of the antigen of interest could realize targeted display on the surface of NPs in a highly repetitive manner. These nanovaccines are highly immunogenic because they mimic most features of pathogens, such as their size, shape, and pathogen-associated molecular patterns (PAMPs) (15, 28, 29). This approach has been shown to improve antigen stability, immunogenicity, and function.

The particulate nature of nanovaccines promotes the uptake of antigens in APCs. The uptake of particles by phagocytosis into phagosomes significantly affects antigen processing as it facilitates cross-presentation of antigen derived epitopes *via* MHC class I and MHC class II pathways (30, 31). Such cross-presentation not only stimulates CD4^+^ Th1 and Th2 cells to induce B cells to initiate antibody responses but Th1 cells can also facilitate differentiation of CD8^+^ T cells towards cytotoxic T cell responses, which is important for protection against intracellular pathogens (32–34). The high antigen density and structurally ordered antigen arrangement on NPs resemble the recognition patterns on pathogens, facilitating the cross-linking of antigens with the BCRs (35). This multivalent interaction is crucial for successful activation of B cells and generation of high levels of neutralizing antibodies (36, 37). These findings highlighted that nanovaccines have multiple advantages over conventional vaccines to induce better immunity and broader protection. However, most of viral protective antigens, such as CSFV E2, fail to form VLP natively, limiting the vaccine efficacy, which leads to the pressing needs for vaccination with NPs.

Previous study has demonstrated computationally designed self-assembling protein mi3 could form icosahedral nanoparticle composed of 60 subunits (38). However, it remains unclear whether this artificial nanoparticle would present similar or even superior potency to native VLPs. In the present study, we constructed recombinant plasmids with CSFV E2 fused to mi3 protein expressed in Bac-to-Bac system. We further characterize the self-assembly efficiency, storage stability and adjuvant effect on APCs of this SP-E2-mi3 NP, in order to evaluate its potential for vaccine development. Further, the SP-E2-mi3 NP-based subunit vaccine was tested against CSFV lethal challenge regarding evoking effective protective efficacy in pigs.

## Materials and Methods

### Cloning

DNA sequences coding for a novel signal peptide (SP) directed truncated CSFV E2 (39), were synthesized according to insect codon usage. The resulting gene was subcloned into baculovirus expression vector pFastBac HTA (Invitrogen, Carlsbad, CA, USA) to generate HTA-SP-E2. mi3 encoding sequences (GenBank AXF54357.1) was then introduced into HTA-SP-E2, with a flexible linker between E2 and mi3 to facilitate proper folding. All the plasmids were constructed in standard methods and verified by DNA sequencing (protein sequences are provided in **Supplementary File 1**). Recombinant baculoviruses were subsequently obtained by Tn7 transposition, bacmid extraction and transfection as described by the manufacturer’s instructions.

### Expression and Purification of Recombinant Protein

Sf-9 cells were pre-seeded in a 500 ml cell culture flask and cultured at 110 rpm, 27°C. When the cell density reached 2–2.5 × 10^6^/ml with the viability over 90%, this dense culture was subject to infection with the recombinant viruses. After another culture for 72–96 h, the whole culture was lysed by ultrasonic disruption, and supernatant containing target proteins was collected and loaded on a HisSep Ni-NTA Agarose Resin (Yeasen, China). The protein was subsequently eluted with PBS buffer (PH 7.4) supplemented with 500 mM imidazole.

After SDS-PAGE separation, protein samples were transferred to PVDF membrane. Blocked with 5% (w/v) non-fat milk for 2 h at room temperature, the membrane was incubated with CSFV E2 monoclonal antibody (3C12) (prepared in our laboratory, 1:2,000 dilution) at 37°C for 1.5 h. After rinses with PBS to remove unbound antibodies, HRP-coupled goat anti-mouse IgG (1:4,000 dilution, Proteintech) was added and incubated at 37°C for 1 h. After three washes, results were visualized by a DAB chromogenic reagent kit (AR1024, Boster, Wuhan, China).

### NP Self-Assembly Physicochemical Conditions and Properties

Purified SP-E2-mi3 fusion protein was filtered using 0.22 μM syringe filter, and subsequently dialyzed against PBS buffer (NaCl 137 mmol/L, KCl 2.7 mmol/L, Na_2_HPO_4_ 4.3 mmol/L, KH_2_PO_4_ 1.4 mmol/L, pH 8.0) at 16°C overnight. Transmission electron microscopy (TEM) was used to evaluate self-assembly of NPs. In brief, 20 µl of sample was loaded onto a copper mesh pre-coated with carbon and allowed to adsorb for 1 min. Subsequently 2% PTA solution (pH 6.8) was added and incubated for 1 min. Excess staining solution was removed by filter paper. The copper meshes were dried in drying oven before the samples were observed by TEM (JEM 1010; JEOL Ltd., Tokyo, Japan). Negative stained samples were analyzed at 80 kV of acceleration voltage and visualized using a Gatan 830 CCD digital Camera (Gatan, CA, USA).

We also evaluated stability to storage at room temperature. SP-E2-mi3 protein was aseptically filtered, adjusted to 0.25 mg/ml divided into small aliquots, and incubated at room temperature. At the indicated time points, the sample was centrifuged to remove aggregates and subject to TEM, then particle number was measured using ImageJ and the Analyze–Analyze Particles tool. To determine the rigidity of the SP-E2-mi3 NPs, dynamic light scattering (DLS) was performed on a Nicomp™ 380 Particle Sizing system (Santa Barbara, CA, USA) at 25°C.

### Cellular Phagocytosis and Cytokine Detection

To verify the optimal concentration of SP-E2-mi3 NPs or SP-E2 for further experiments, the cytotoxic effect of these proteins on PAM/3D4 cells was evaluated by MTT cell viability assay (Thermo Scientific Inc.) as described in the user manual.

FITC-labeled SP-E2 (FITC-SP-E2) and SP-E2-mi3 NPs (FITC-SP-E2-mi3 NPs) were prepared using a commercialized kit (Sangon, China) according to the user manual. Internalization of SP-E2-mi3 NPs by PAM/3D4 cells was determined as following steps. In brief, cells were pre-seeded on 96-well cell culture plates at 2 × 10^5^ cells/ml and cultured overnight. FITC-SP-E2 or FITC-SP-E2-mi3 NPs were diluted by NP assembly solution and adjusted to the same molality (100 μM). Approximately 100 μl of FITC-SP-E2 or FITC-SP-E2-mi3 NPs were then added and incubated at 37°C for 2 h. DAPI solution (1 μg/ml) was used to stain cell nuclei for 5 min before immunofluorescence signals corresponding to antigen SP-E2 internalization were detected by microscopy. The number of FITC positive cells and DAPI positive cells (total viable cells) were respectively counted. The internalization rate of SP-E2 was read as (the number of FITC positive cells)/(the number of DAPI positive cells) × 100%.

The surface binding and internalization of SP-E2-mi3 NPs into PAMS was also recorded by a confocal laser scanning microscopy. The amount of 100 μM of FITC-SP-E2 or FITC-SP-E2-mi3 NPs was added which was incubated at 37°C for 2 h. The late endosomes and lysosomes were stained with 75 nM LysoTracker Red DND-99 probe (Yeasen, Shanghai) for 30 min before collecting fluorescent images by a confocal laser scanning microscopy IX81-FV1000 (Olympus).

After incubation with SP-E2-mi3 NPs or SP-E2, the total RNAs were isolated from PAM/3D4 cells using an Easy RNA Kit (Zhejiang Easy-Do Biotech CO., LTD) as described by the manufacturer. cDNA was synthesized by HiScript^®^ II Reverse Transcriptase (Vazyme, Nanjing, China) according to the user manual. The primer sequences for quantitative polymerase chain reaction (qPCR) were as follows: TNF-α, forward primer, 5’-ACT CGG AAC CTC ATG GAC AG-3’, reverse primer, 5’-GGG GTG AGT CAG TGT GAC C-3’; IL-12, forward primer, 5’-CCA TTG AGG TCG TGC TGG AA-3’, reverse primer, 5’-TGC CCT GAA CTT GAA CAC CA-3’; GAPDH, forward primer, 5’-TTC CGT GTC CCT ACT GCC AAC-3’, reverse primer, 5’-ACG CCT GCT TCA CCA CCT TCT-3’. GAPDH was used as an internal reference to normalize mRNA expression. The relative expression was represented as fold changes using 2^−ΔΔCt^ method (40).

### Immunization and Challenge Study

Vaccines were prepared by emulsifying antigens (SP-E2-mi3 NPs, SP-E2 or PBS) with ISA-206 adjuvant (Seppic, France) (1:1, w/w) according to the manufacturer’s manual. For prime-boost immunization schedule, sixteen 4-week-old piglets without CSFV, PCV2 and PRRSV, were randomly divided into four groups with four animals per group. Groups A and B were intramuscularly inoculated with 10 μg SP-E2-mi3 NPs or 10 μg SP-E2, respectively. PBS immunized Group C served as a negative control. Group D was blank control without any treatment. Booster immunization was given by the same dose and administration route at 21 days post-immunization (dpi). All the vaccinated piglets were challenged intramuscularly with 10^5^ TCID_50_ CSFV Shimen strain at 35 dpi.

For single dose immunization schedule, twelve 4-week-old pigs were randomly divided into three groups (A, B and C, n = 4), and subject to an immunization and challenge trial. In brief, Groups A and B were respectively immunized with 10 μg SP-E2-mi3 NPs or PBS intramuscularly. Group C was left untreated. All the vaccinated pigs were challenged intramuscularly with 10^5^ TCID_50_ CSFV Shimen strain at 28 dpi. At different time points, whole blood samples and serum samples were collected for further analysis. All experiments with live viruses including viral propagation, titration and *in vivo* challenge, were conducted in biosafety facilities according to the guidelines of the OIE manuals (41).

Rectal temperatures and clinical scores were used to determine the health status of the pigs following the established standards with modifications (42). Clinical scores were evaluated based on five clinical parameters, including body tension, walking, appetite, defecation and eyes/conjunctiva (normal, 0; slight, 1 point; distinct, 2; severe, 3). The observed parameters were collected daily.

### Serological Assays

Porcine serum samples were collected at 0, 7, 21, 28 and 35 dpi. CSFV E2-specific antibody was determined using a commercial ELISA kit (IDEXX Laboratories, Shiphol-Rijk, The Netherlands) according to the user manual. Virus neutralizing antibody was tested by a serum-virus neutralization test (SNT) according to other related researches (39, 43).

### CSFV-Specific IFN-γ Enzyme-Linked Immunospot Assay

Peripheral blood mononuclear cells (PBMC) were obtained from anticoagulant blood samples collected at 21 and 35 dpi. This isolation method based on density gradient was performed using a commercial product (P8770, Solarbio, Beijing, China) according to the user manual. Specific cellular responses against CSFV were measured by a Porcine IFN-γ ELISpot PLUS (ALP) kit (Mabtech AB, Nacka Strand, Sweden) as described by the manufacturer. In brief, porcine IFN-γ mAb coated plates were blocked with PBMC medium supplemented with 10% FBS for 30 min at 25°C. After double washes to remove residual medium, 10^5^ PBMCs and 10^4^ TCID_50_ CSFV Shimen strain were simultaneously added and allowed to incubate for another 36 h to stimulate the secretion of IFN-γ. Cell culture medium was discarded, followed by rinsing five times with PBS buffer. Subsequently, the biotinylated mAb P2C11 was added at a final concentration of 0.5 μg/ml. After 2 h incubation, plates were incubated with streptavidin-ALP (1:1,000 diluted in PBS buffer supplemented with 0.5% FBS) for 1 h at 25°C. IFN-γ specific spots were developed by BCIP/NBT substrate. Spot counting was performed by an automatic EliSpot Reader (AID Diagnostika, Strassberg, Germany).

### Viremia and Leukopenia Assessment

Anticoagulant whole blood samples were collected at 3, 6, 9, 12 and 15 dpc, and total RNA was isolated and converted to cDNA as described above. CSFV RNA loads were detected by qPCR a primer pair (5′-CTC CCA GCA CGT GGT GTG ATT TC-3′ and 5′-TGG GTG GTC TAA GTC CTG AGT A-3′), corresponding to 5’ UTR of the viral genome. Leucocyte counting was calculated by a Mindray BC-30 automated hematology system (Mindray Medical USA Corp, Mahwah, USA).

### Pathological and Microscopic Lesion Evaluation

All the survived pigs were humanely euthanized and autopsied at 15 days post-challenge (dpc). A variety of tissue samples, including inguinal lymph nodes, submandibular lymphatic nodes, kidney, and spleen, were collected and fixed for hematoxylin–eosin staining (H&E) and immunohistochemistry (IHC) assay according to another related research (39).

### Statistical Analysis

The data analysis was performed by Student’s t test when two groups were compared, or by one-way analysis of variance (ANOVA) for more than two groups where P <0.05 was considered statistically significant.

## Results

### Preparation and Characterization of CSFV E2-Based Nanovaccine

**Figure 1:**
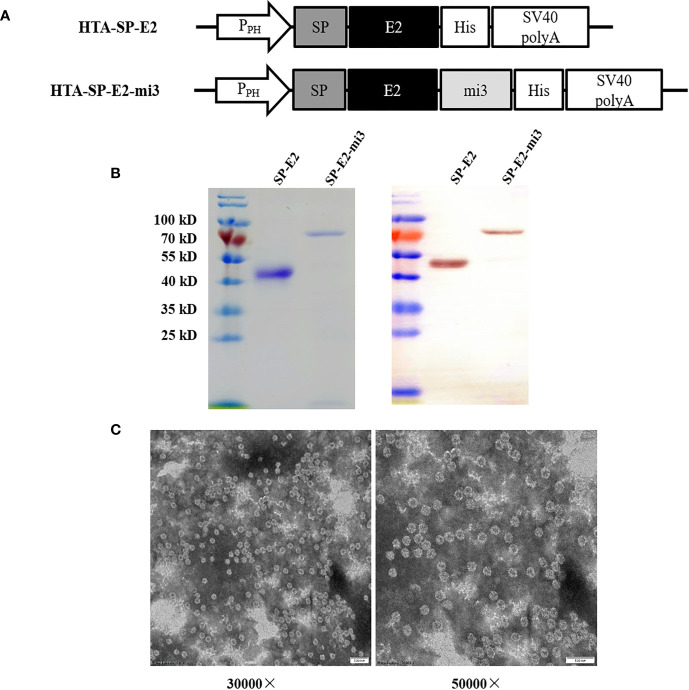
Expression and self-assembly analysis of mi3 fusion proteins. **(A)** Schematic diagram of baculoviral constructs for eukaryotic expression. P_PH_: AcMNPV polyhedrin promoter, mi3: self-assembling protein, SV40 polyA: SV40 polyadenylation signal. **(B)** Analysis of purified SP-E2 and SP-E2-mi3 proteins in SDS-PAGE (left) and Western blot (right). The target proteins were detected with E2 monoclonal antibody 3C12. **(C)** TEM analysis of self-assembly capacity of SP-E2-mi3 fusion protein.

To investigate the self-assembly and the antigenicity of the SP-E2-mi3 protein, SP-E2 and SP-E2-mi3 expression plasmids were generated as shown in **Figure 1A**. The fusion proteins were successfully overexpressed in Sf9 cells and subject to one-step purification using 12× His tag mediated metal affinity chromatography under native conditions. As shown in **Figure 1B**, all the protein bands were consistent with their predicted sizes. SP-E2-mi3 and SP-E2 were obtained with the purity above 90%, and developed considerable reactivity with E2 monoclonal antibody 3C12.

**Figure 2:**
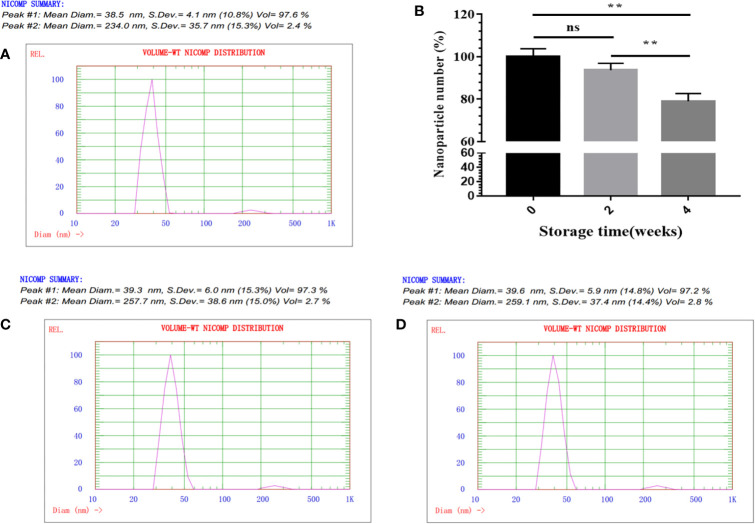
physicochemical characterization of SP-E2-mi3 NPs. **(A)** Hydrodynamic diameter and size distribution of mi3 NPs was analyzed by DLS. **(B)** Storage stability analysis of SP-E2-mi3 NPs. SP-E2-mi3 NPs was stored at room temperature for 0, 2 and 4 weeks, and then subject to NPs imaging by TEM. NPs counting were calculated by ImageJ. DLS was then performed to show size distribution of SP-E2-mi3 NPs stored for 2 **(C)** and 4 weeks **(D)**. All analyses were performed in triplicate. (**p < 0.01; ns, not significant, p > 0.05).

Next, NP assembly was carried out *via* buffer exchange to PBS and examined by TEM. The results showed that fusion protein SP-E2-mi3 could self-assemble into uniform NPs, which had an approximate diameter of 25–35 nm (**Figure 1C**). The hydrodynamic size was then determined by DLS. The size distribution of SP-E2-mi3 NPs was composed of two types, among which 97.6% are 38.5 nm and the remaining 2.4% are 234 nm (**Figure 2A**). As DLS gives a hydrodynamic size in the solvent different from TEM analysis in a dried state (26), most of NPs exhibited similar diameter to that of TEM analysis.

### Stability Analysis

For an effective vaccine, storage stability is an important parameter. Next, the storage stability was evaluated at room temperature. The percentage of intact SP-E2-mi3 NPs is more than 90% for 2 weeks, and ~80% of NPs are still preserved for 4 weeks (**Figure 2B**). Besides the solubility, it was interesting to explore whether the remaining NPs could maintain a narrow size distribution. 97.3% for 2 weeks (**Figure 2C**) and 97.2% for 4 weeks (**Figure 2D**) of NPs gave a similar diameter to the initial state (**Figure 2A**). Taken together, SP-E2-mi3 NPs exhibited adequate particle size uniformity and storage stability at room temperature.

### Cellular Uptake and Cytokine Induction With SP-E2-mi3 NPs

**Figure 3:**
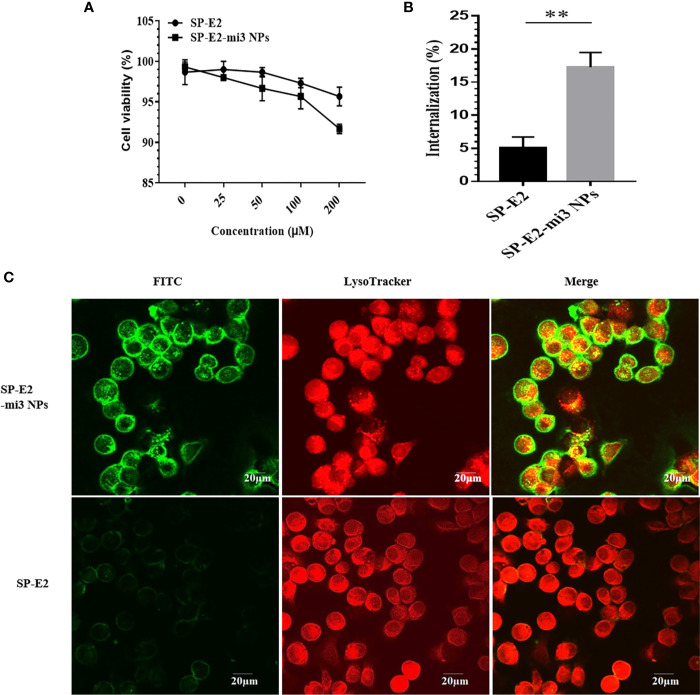
Evaluation of internalization efficiency by PAM/3D4 cells. **(A)** Cytotoxicity of SP-E2-mi3 NPs or SP-E2 on PAM/3D4 cells at various concentrations after incubation for 24 h. **(B)** PAM/3D4 cells were incubated with FITC-SP-E2 or FITC-SP-E2-mi3 NPs for 2 h. The internalization efficiency of SP-E2 was determined, and all results were presented from triplicate experiments. NPs counting were calculated by ImageJ. All analyses were performed in triplicate. **(C)** After exposed to FITC-SP-E2 or FITC-SP-E2-mi3 NPs for 2 h, the binding and internalization of SP-E2-mi3 NPs was analyzed. Signals of SP-E2 or SP-E2-mi3 NPs (green) and LysoTracker (red) were visualized with confocal microscopy. **p < 0.01.

Antigen uptake by immune cells is the initial step in the generation of robust adaptive immunity. Porcine alveolar macrophage line (PAM/3D4), as a model, is widely used in the study of swine viral diseases. The cytotoxicity of SP-E2-mi3 NPs was firstly assessed in PAM/3D4 cells. As expected, both SP-E2-mi3 NPs and SP-E2 did not induce any significant decrease in cell viability even at concentration up to 100 μM. Cell viability sharply decreased to less than 90% at the concentration of 200 μM with SP-E2-mi3 NPs, but cell viability remained above 95% for 200 μM of SP-E2 (**Figure 3A**). Compared with SP-E2, the decrease in cell activity with 200 μM of SP-E2-mi3 NP may be caused by the partial aggregation of nanoparticles on cell surface after prolonged incubation.

Subsequently, we quantified the internalization efficiency of SP-E2-mi3 NPs or monomer SP-E2 in PAMs. It is well-known that NPs of nanoscale sizes similar to pathogens (10–200 nm) are preferred to uptake by APC. In contrast to soluble antigens, NP-delivered antigens developed enhanced uptake and internalization by APCs (31). Similarly, uniform SP-E2-mi3 NPs (~30 nm in diameter) incubated cells developed fluorescence intensity, as compared to the cells incubated with monomer SP-E2. More E2 antigens were internalized when the antigen was delivered in the term of SP-E2-mi3 NPs. The internalization efficiency of SP-E2 was determined, at 17.26% for SP-E2-mi3 NPs and only 4.9% for SP-E2, with significant difference (**Figure 3B**, P < 0.01).

Next, the internalization and cellular localization of FITC-SP-E2-mi3 NPs or FITC-SP-E2 were examined by confocal laser scanning microscopy. Compared to monomer SP-E2, SP-E2-mi3 NPs developed stronger fluorescence signals, which indicated improved antigen binding and internalization in PAMs (P < 0.01) (**Figure 3C**).

**Figure 4:**
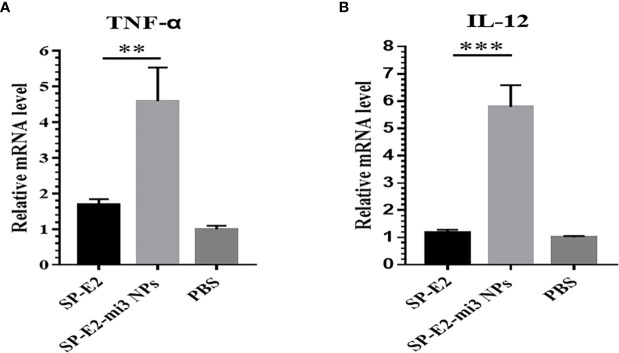
Antigen internalization induced immunostimulatory effects in *vitro*. After exposed to FITC-SP-E2 or FITC-SP-E2-mi3 NPs for 2 h, total RNAs were isolated from stimulated PAMs, and mRNA levels TNF-α **(A)** and IL-12 **(B)** were determined in qPCR, and the data were presented from triplicate experiments. **p < 0.01; ***p < 0.001.

It is well established that Th1-polarizing cytokines TNF-α and IL-12 secreted by mature APCs, are responsible for promoting T cell proliferation and cytokines production, which play critical roles in eliciting protective cellular immune responses (13). SP-E2-mi3 NPs instead of monomer SP-E2 stimulated high transcription level of TNF-α and IL-12 (**Figures 4A, B**). From these results, we conclude that mi3 NPs may act as a strong T cell adjuvant in promoting CD8^+^ T cells activation and CTL responses.

### Enhanced Immune Response Induced by SP-E2-mi3 Nanovaccines in Pigs

**Figure 5:**
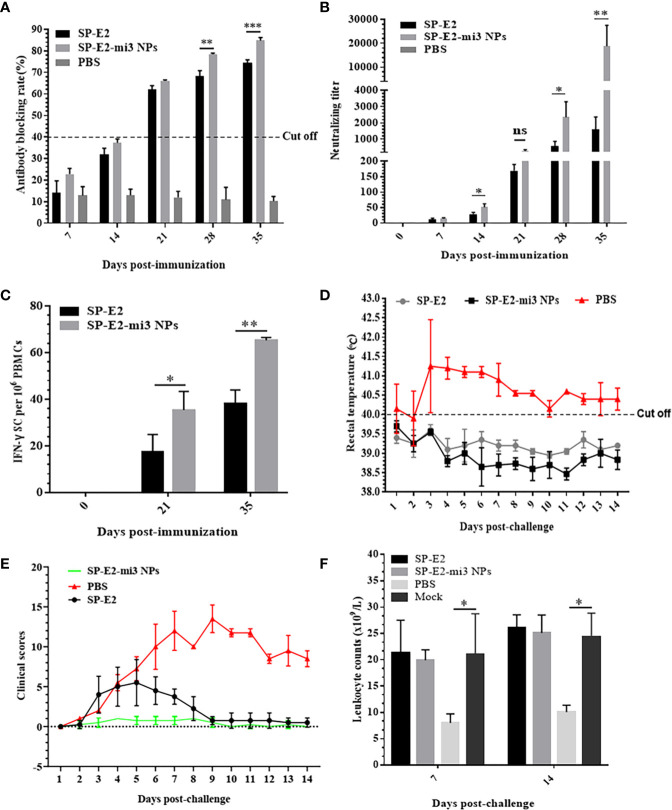
Protective efficacy of SP-E2-mi3 NPs in pigs. Three groups (n = 4) of 4-week-old pigs were intramuscularly inoculated with 10 μg SP-E2-mi3 NPs, 10 μg SP-E2 and PBS, respectively. The remaining group was left untreated. **(A)** Detection of CSFV-specific antibodies in serum samples collected at the indicated time points using the IDEXX HerdChek^®^ CSFV Antibody Test Kit. **(B)** Detection of CSFV neutralizing antibody in pig serum samples. Neutralization titer was determined against CSFV Shimen strain. **(C)** Detection of CSFV-specific IFN-γ secreting cell responses against CSFV Shimen strain in vaccinated pigs. **(D)** Rectal temperatures of the pigs following CSFV Shimen strain challenge. **(E)** Clinical scores were evaluated based on five CSFV-induced typical clinical symptoms, and the data was collected daily. **(F)** Leukopenia assessment in vaccinated pigs following CSFV Shimen strain challenge. Mock indicated the untreated healthy pigs. ns, not significant, p > 0.05; *p < 0.05; **p < 0.01; ***p < 0.001

The efficacy of SP-E2-mi3 NPs to induce *in vivo* protective response was further investigated in pigs. No significant side effects were observed following immunization. As mi3 is derived from artificial proteinaceous self-assembling molecule, it may not cause any side effects or safety concerns (38). Specific antibodies and neutralizing antibodies against CSFV were evaluated by blocking ELISA and SNT. The PBS vaccinated group only induced non-specific antibody response. Both the SP-E2-mi3 NPs and SP-E2 vaccinated group developed positive antibody levels (cut-off value = 40%) from 21 dpi, and the blocking rate reached the highest level at 35 dpi, with 84.8% in SP-E2-mi3 NPs vaccinated group, 74.3% in SP-E2 vaccinated group. SP-E2-mi3 NPs generated significantly higher specific antibody levels than SP-E2, with significant difference at 28 dpi (P <0.01) and 35 dpi (P <0.001) (**Figure 5A**).

In previous research, homologous subunit vaccine immunization is effective to elicit a predominant humoral immune response, but not induce adequate cell-mediated immune response, which is crucial for protection against intracellular pathogens (23, 44–46). Hence, neutralizing antibody levels were determined, which are related to *in vivo* protective efficacy. Sera collected at 0, 7, 14, 21, 28 and 35 dpi were subjected to SNT. At 7 dpi, neutralizing antibodies against CSFV were detectable in both groups of SP-E2-mi3 NP and SP-E2, and the mean neutralizing antibody titers were 1:14 and 1:11, respectively. The neutralizing antibody titers reached a peak at 35 dpi (2 weeks after booster immunization), with 1:17,560 in SP-E2-mi3 NP vaccinated group, 1:1,448 in SP-E2 vaccinated group. Significant difference was detected in neutralizing antibodies against CSFV between SP-E2-mi3 NP and SP-E2 vaccinated group at 14 dpi and 28 dpi (P <0.05), especially at 35 dpi (P <0.01). None of the PBS vaccinated group developed any detectable neutralizing antibodies against CSFV (**Figure 5B**).

IFN-γ secreted by Th1 cells plays critical roles in regulating the cell-mediated immunity, which reflect the antiviral activity of the host. Both vaccinated groups had obvious numbers of CSFV-specific IFN-γ secreting cells in PBMCs compared to PBS vaccinated group. SP-E2-mi3 NP vaccinated group stimulated significantly increased IFN-γ secreting cells than that of SP-E2 vaccinated group, for 21 dpi (P <0.05) and 35 dpi (P <0.01) (**Figure 5C**).

### Protective Efficacy Against CSFV Lethal Challenge Stimulated by SP-E2-mi3 Nanovaccine

All the features of nanovaccine described above contribute to *in vivo* protection against CSFV in pigs. Following CSFV challenge, the PBS vaccinated group exhibited acute fever (40.5–42.1°C) (**Figure 5D**) and other CSFV-induced typical clinical symptoms include mild diarrhea, chill, loss of appetite, prostration and conjunctivitis from 3 dpc till the end. In SP-E2 vaccinated group, two pigs developed loss of appetite, diarrhea and conjunctivitis, and recovered from 3 to 9 dpc. No febrile response and other clinical symptoms were observed in SP-E2-mi3 NP vaccinated group (**Figure 5E**).

Both vaccinated groups did not present viremia. However, high-level viral RNA was detectable in unimmunized group upon challenge, with 10^4^ copies/μl at 6 dpc, and reached a peak at 15 dpc (10^5^ copies/μl) (**Table 1**). Corresponding to the viremia, leukopenia was also detected at 7 and 14 dpc. Compared to the uninfected group, leukocyte counts were significantly down-regulated in challenged group without immunization (PBS vaccinated group). But the leukocyte counts in both immunized groups did not show significant change upon challenge, indicating that the groups stay healthy just like mock-challenge group (**Figure 5F**).

**Table 1:** Detection of viral RNA in the whole blood samples from the SP-E2-mi3 NPs vaccinated piglets by qPCR.

Group	Viral RNA(copies/μl)				
	3 dpc	6 dpc	9 dpc	12 dpc	15 dpc
SP-E2-mi3 NPs	/	/	/	/	/
SP-E2	/	/	/	/	/
PBS	/	(12.4 ± 2.72)×10^3^	(26.4 ± 4.45)×10^3^	(16.7 ± 9.95)×10^4^	(22.3 ± 8.45)×10^4^

Three groups (n = 4) of 4-week-old piglets immunized with SP-E2-mi3 NPs, SP-E2 or PBS were challenged with the highly virulent CSFV Shimen strain. Anticoagulant whole blood samples were collected at 3, 6, 9, 12 and 15 dpc, CSFV RNA loads were detected by qPCR./, undetectable.

**Figure 6:**
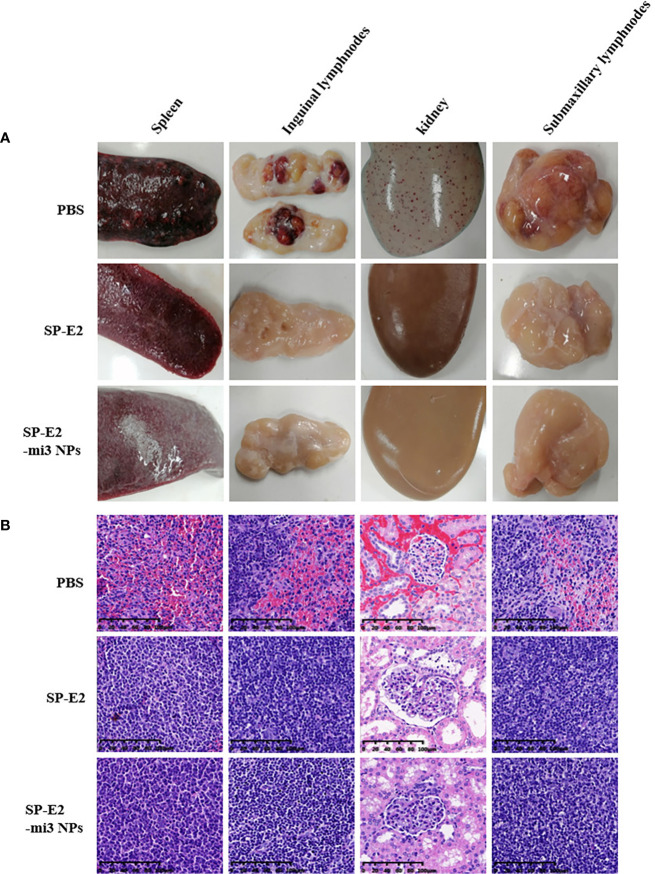
Representative pathological and histopathological examination of vaccinated pigs upon CSFV lethal challenge. A variety of tissue samples, including inguinal lymph nodes, submandibular lymphatic nodes, kidney, and spleen, were collected and subject to pathological observation **(A)** and histopathological examination using hematoxylin–eosin staining (H&E) staining **(B)**.

The protective efficiency was also supported by pathological and histopathological analysis. Pathological analysis revealed SP-E2-mi3 NP vaccinated group did not show any changes in different tissues and organs. There was infarct at the margin of the spleen in SP-E2 vaccinated group. PBS vaccinated group exhibited severe clinical lesions. These include extensive hemorrhage in the kidney, lymph node enlargement and hemorrhage, and splenic infarction and petechia (**Figure 6A**).

**Figure 7:**
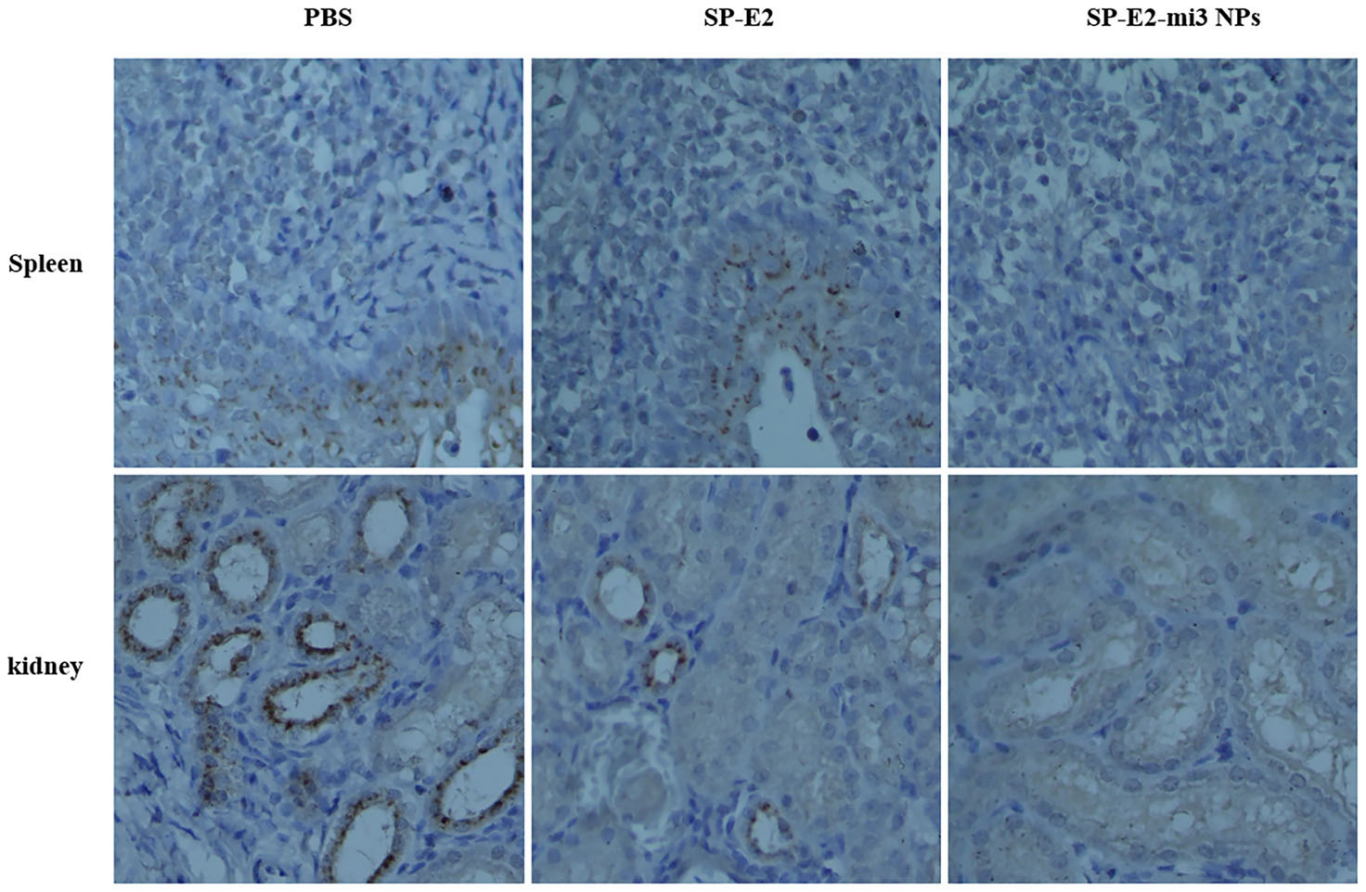
Detection of CSFV antigen in the tissues from SP-E2-mi3 NPs vaccinated piglets by immunohistochemistry assay. Using CSFV polyserum as primary antibody, the brown dots represent CSFV specific signals.

Also, no histopathological changes were detected in SP-E2-mi3 NP vaccinated group. In line with splenic lesions, sporadic petechiae were detected in the spleen for SP-E2 vaccinated group. In contrast, in PBS vaccinated group, some tissues of pigs showed severe histopathological changes, such as significant lymphocytosis and massive parenchymal hemorrhage in lymph nodes, extensive bleeding in the spleen and significant lymphocytosis in splenic white pulp, and numerous bleeding points in kidney (**Figure 6B**). In **Figure 7**, IHC assay revealed that distinct CSFV signals (brown) were detected in tissues of PBS vaccinated group, and slightly less CSFV signals were also observed in SP-E2 vaccinated group. However, no CSFV positive signals were observed in tissues of SP-E2-mi3 NP vaccinated group. Based on these results, it can be concluded that in a prime-boost immunization schedule, 10 μg SP-E2-mi3 NPs instead of SP-E2 conferred complete protection against CSFV lethal challenge.

### Clinical Protection Elicited by Single Dose of SP-E2-mi3 NPs Upon Lethal Challenge

**Figure 8:**
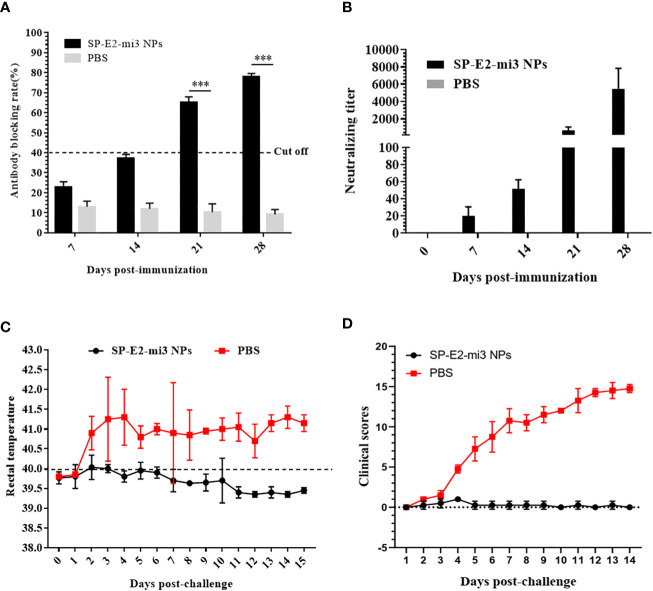
Protective efficacy elicited by single dose of SP-E2-mi3 NPs in pigs. three groups (n = 4) of 4-week-old piglets were intramuscularly inoculated with 10 μg SP-E2-mi3 NPs, 10 μg SP-E2 and PBS, respectively. The remaining group was left untreated. **(A)** Detection of CSFV-specific antibodies in serum samples collected at the indicated time points using the IDEXX HerdChek^®^ CSFV Antibody Test Kit. **(B)** Detection of CSFV neutralizing antibody in pig serum samples. Neutralization titer was determined against CSFV Shimen strain. **(C)** Rectal temperatures of the piglets following CSFV lethal challenge. **(D)** Clinical scores were evaluated based on five CSFV-induced typical clinical symptoms, and the data was collected daily. ***p < 0.001.

As previous studies have reported incomplete prevention from lethal challenge using a single vaccination of soluble E2 protein vaccines (2, 11), the efficacy of a single dose of SP-E2-mi3 NPs was evaluated here against CSFV. No significant side effects were observed following vaccination. Specific antibodies and neutralizing antibodies against CSFV were determined as mentioned above. SP-E2-mi3 NPs vaccinated group developed positive antibody levels (cut-off value = 40%) from 21 dpi, and the blocking rate reached the highest level (77.9%) at 35 dpi. The PBS vaccinated group only induced a non-specific antibody level below the cut off line (**Figure 8A**). At 7 dpi, neutralizing antibodies against CSFV were detectable in SP-E2-mi3 NPs vaccinated group, and the mean neutralizing antibody titers were 1:18. The neutralizing antibody titers reached a peak at 28 dpi, with 1:5,042 in SP-E2-mi3 NPs vaccinated group. None of the PBS vaccinated group developed any detectable neutralizing antibodies against CSFV (**Figure 8B**).

The PBS vaccinated group exhibited acute fever (40.7–42°C) and other CSFV-induced typical clinical symptoms include mild diarrhea, chill, loss of appetite, prostration and conjunctivitis from 3 dpc till the end. Besides, one pig died at 12 dpc. No febrile response and other clinical symptoms were observed in SP-E2-mi3 NP vaccinated group (**Figures 8C, D**). Whole blood samples were collected at 3, 6, 9 and 12 dpc, and viral RNA was also detected by qPCR. CSFV RNA loads exceeding 10^3^ copies/μl were detected in PBS vaccinated group, and peaked at 12 dpc (>10^5^ copies/μl) (**Table 2**). CSFV RNA was detected in two out of four pigs for SP-E2-mi3 NPs vaccinated group at 6 and 9 dpc, with RNA loads of <10^4^ copies/μl. Nevertheless, no CSFV RNA was detectable at 12 dpc in all pigs of in SP-E2-mi3 NP vaccinated group. These findings indicated that 10 μg of SP-E2-mi3 NP vaccinated piglets developed high level specific antibodies and neutralizing antibodies, which was able to reduce clinical symptoms and viremia, but only provide clinical protection against lethal virus challenge.

**Table 2:** Detection of viral RNA in the whole blood samples from single dose of SP-E2-mi3 NPs vaccinated piglets by qPCR.

Group	Pig No.	Viral RNA(copies/μl)			
		3 dpc	6 dpc	9 dpc	12 dpc
SP-E2-mi3 NPs	4	/	/	/	/
	21	/	2.24 × 10^2^	/	/
	24	/	4.17 × 10^3^	8.72 × 10^2^	/
	55	/	/	/	/
PBS	34	1.24 × 10^3^	3.21 × 10^4^	3.35 × 10^5^	3.57 × 10^5^
	38	8.92 × 10^3^	7.85 × 10^5^	1.08 × 10^6^	–
	57	3.56 × 10^3^	8.96 × 10^4^	7.46 × 10^5^	1.43 × 10^6^
	89	3.12 × 10^3^	5.93 × 10^4^	6.25 × 10^5^	8.66 × 10^5^

Two groups (n = 4) of 4-week-old piglets immunized with SP-E2-mi3 NPs or PBS were challenged with the highly virulent CSFV Shimen strain. Anticoagulant whole blood samples were collected at 3, 6, 9 and 12 dpc, CSFV RNA loads were detected by qPCR./, undetectable; –, died.

## Discussion

Although the widespread application of C-strain attenuated vaccine has suppressed the endemic outbreak to a great extent, CSFV is still spreading and circulating in many countries including China. Glycoprotein E2-based DIVA vaccines, including subunit, DNA, and viral vector vaccines (10, 11, 47–49), are faced with various challenges in biosafety and efficacy, thereby hampering CSF eradication. Therefore, it is necessary to develop a broadly effective, safe and reliable DIVA vaccine.

Multiple nanotechnology platforms such as VLPs, self-assembling protein nanoparticles, biodegradable polymers and liposomes have been investigated for vaccine delivery. Self-assembling protein nanoparticles have shown several benefits over other nano-structured systems used for vaccine development, and their usage in vaccine design is emerging based on their advantages of multivalency, biodegradability, biocompatibility (15, 28). Thus, this strategy enables the design of nanovaccines to augment vaccine stability and immunogenicity towards improved protective immunity. However, no studies have investigated the effect of self-assembling protein nanoparticles carrying a target antigen against CSFV.

Here, we utilized the self-assembling peptide mi3 to form a CSFV E2-based nanovaccine. Following one-step purification, SP-E2-mi3 fused protein was found to fold correctly and self-assemble into uniform NPs *in vitro*. Furthermore, in view of the fact that previously solved structure of mi3 NPs revealed that N terminal of mi3 monomer was exposed to outer surface (38), N terminal fused E2 would be favorable for surface display on SP-E2-mi3 NPs.

It is notable that cold chain spends about 80% of vaccine production cost (50, 51). Generation of vaccines with excellent storage stability is an essential step to accelerate massive vaccination in developing countries (25, 52). In the present study, self-assembling SP-E2-mi3 NPs exhibited considerable thermostability. Even at room temperature for 4 weeks, most NPs possessed the nanoscale properties with the loss of 20% due to aggregation. Actually, several previous reports indicated that Enterovirus 71 (EV71) and type 2 porcine circovirus (PCV2) VLPs had stable morphology (shape and size distribution) in optimized buffer after storage for 1 month at 25°C (53, 54). It was found the *in vitro* antigenicity was highly related to homogeneous integrity. In the present study, mi3 NPs maintained a narrow size distribution after storage at 25°C for 4 weeks, which was consistent to the previously reported stability. Therefore, it was concluded that these intact NPs retained the original antigenicity and biological activity, which make them ideal tools for vaccinological and therapeutic applications (25, 55).

Emerging studies have indicated that NPs show enhanced adsorption and phagocytosis by APCs (DCs and macrophages), and stimulate their maturation, including the up-regulation of major histocompatibility complex classes I and II, CD86, CD80 and cytokine production (56). Accordingly, SP-E2-mi3 NPs promoted antigen binding and internalization into PAMs, which may be attributed to the fact that mi3 NPs enable multiple adsorption and delivery of SP-E2. Transcription levels of immunostimulatory cytokines TNF-α and IL-12 were also significantly up-regulated, which are believed to facilitate cross-presentation of antigen and enhance protective immunity (31, 57). Our data validated the ability of this NP-based vaccine for elevated antigen uptake, processing and presentation.

Most previous studies use model proteins (OVA) and mice as animal model to explore the feasibility of vaccine. Here, we are making efforts to generate efficient vaccines for large animals in the field. Considering that SP-E2-mi3 NPs possessed promising properties as a vaccine *in vitro*, we therefore evaluated the *in vivo* protection of the SP-E2-mi3 NPs through an immunization and challenge trial. As expected, SP-E2-mi3 NPs were well related to humoral and cellular immune responses, especially the elevated CSFV-specific IFN-γ secreting cells and >10-fold neutralizing antibodies at 35 dpi, which represent a significant improvement of protective immune responses in the elimination of intracellular pathogens. High titres of neutralizing antibodies play a relevant role in protection against highly virulent CSFV strains. Moreover, CSFV-specific IFN-γ confers protection against CSFV even in the absence of neutralizing antibodies a few days after vaccination (58, 59). This finding provided supporting evidence that NP-based strategy offers great advantages over rational subunit vaccines, and NPs can induce a higher level of immune response compared to monomeric antigens (60, 61).

Actually, these significantly increased factors correlate well with *in vivo* protection against CSFV. Following lethal dose of CSFV challenge, no CSFV-related clinical signs, viremia and leukopenia were detected in SP-E2-mi3 NPs vaccinated group, and these situations were supported by histopathological observation. By contrast, SP-E2 vaccinated group exhibited some typical clinical signs and splenic lesions. These results supported that SP-E2-mi3 NPs as an effective nanovaccine could offer complete protection against CSFV. It was consistent with previous findings that self-assembling protein nanoparticles allowed targeted display of antigens and mimicked the natural conformation of pathogens, resulting in improved and extended vaccine efficacy against multiple diseases (15, 37).

Subunit vaccines mostly offer homologous prime-boost schedules, in which priming and boost were given by the same dose and administration route (62). Multiple immunizations and high antigen dosage are employed in most subunit vaccines, and it may increase the cost of vaccination, especially for large animal. Thus, we followed up with a single dose of 10 μg of SP-E2-mi3 NPs. 10 μg of SP-E2-mi3 NPs vaccinated piglets developed high level specific antibodies and neutralizing antibodies. Most importantly, single dose of SP-E2-mi3 NPs elicited markedly higher level of neutralizing antibodies (1:5,042 at 28 hpi, 2 weeks after prime immunization), whereas 10 μg of SP-E2 only stimulated neutralizing antibody titers 1:1,448 at 35 hpi (2 weeks after booster immunization). The difference of ~3.5-fold in neutralizing antibodies highlighted potential applications of NPs in vaccine development. These findings suggested that NPs-based delivery strategy could overcome the drawbacks of conventional subunit vaccines, such as poor cell-mediated immunity and multiple doses. Following CSFV challenge, PBS vaccinated group showed CSFV-related clinical symptoms with typical fever and prostration, and one pig died at 12 dpc. However, a single dose of 10 μg of SP-E2-mi3 NPs reduced clinical symptoms and viremia, but further optimization is required to provide full protection. In view of the fact that the SP-E2-mi3 NPs was initially tested to evaluate protective potency in pigs, it can be further improved for better efficacy in terms of the minimal effective dose, or the employment of optimized adjuvant specialized for most licensed VLP-based vaccines (63, 64).

It remains controversial whether a difference in infectivity would occur between intramuscular and intranasal infections of CSFV (65, 66). Many studies used an intramuscular route to confirm the exact same dose of viral infection (47, 67–69). Also, other studies adopted oronasal challenge route to mimic natural infection by using delivery methods (65, 70, 71). It is noted that for oronasal challenge route, specialized nasal drug aerosol delivery devices are required to ensure the exact same dose of viral infection to the maximum. Oronasal challenge route is actually similar to natural infection in terms of pathogenicity, dynamic distribution and tissue tropism. To really highlight the ability of the nanoparticle formulations to induce better responses important for protection against the natural route of infection, it is necessary to explore oronasal challenge route instead of intramuscular route in future studies.

In conclusion, we proposed a novel strategy to develop vaccines by displaying antigen on genetically encoded NPs. The target antigen and the self-assembling peptide scaffold are simply combined as a fusion protein, forming stable NPs carrying CSFV E2. SP-E2-mi3 NPs possessed adequate storage stability to room temperature and maintained a narrow size distribution, thereby reducing the vaccine cost to accelerate massive vaccination in developing countries where cold chain is not easily available. Furthermore, we have shown that CSFV E2 vaccine made with this technology elicited enhanced antigen processing and presentation by APCs *via* up-regulated internalization and transcriptional level of immunostimulatory cytokines. Importantly, in a large animal model against CSFV lethal challenge, the nanovaccine significantly improved protective efficacy compared to monomeric E2. For the first time, the study not only elucidated the mechanisms for the improved efficacy but also fulfilled the vaccine trials in a practical veterinary model, which accelerates the application of self-assembling nanovaccines as compared to conventional studies with standard proteins (OVA) and mice. Hence, the findings provide valuable guidance for novel antiviral strategies in the current global background against infectious agents.

## Data Availability Statement

The datasets presented in this study can be found in online repositories. The names of the repository/repositories and accession number(s) can be found in the article/**Supplementary Material**.

## Ethics Statement

The animal study was reviewed and approved by the Laboratory Animal Management Committee of Zhejiang University (Approval No. 2019028).

## Author Contributions

Z-HL was responsible for sample collection, experiment performance, analyzed the data and manuscript drafting. H-LX and G-WH performed some of the experiments and data analysis. L-NT, YL, and S-YZ provided suggestions and proofread the manuscript. W-HF provided experimental materials and technical guidance. FH designed the study, analyzed the data and proofread the manuscript. All authors contributed to the article and approved the submitted version.

## Funding

This work was supported by Zhejiang Province Key R&D Program (Novel CSFV vaccine research and development, No. 2021C02051) and 100 Talent Program of Zhejiang University.

## Conflict of Interest

The authors declare that the research was conducted in the absence of any commercial or financial relationships that could be construed as a potential conflict of interest.

## References

[1] Edwards S Fukusho A Lefèvre PC Lipowski A Pejsak Z Roehe P. Classical Swine Fever: The Global Situation. Vet Microbiol (2000) 73:103–19. 10.1016/S0378-1135(00)00138-3 10785321

[2] Blome S Staubach C Henke J Carlson J Beer M. Classical Swine Fever-An Updated Review. Viruses (2017) 9:86. 10.3390/v9040086 PMC5408692 28430168

[3] Gong W Li J Wang Z Sun J Mi S Lu Z. Virulence Evaluation of Classical Swine Fever Virus Subgenotype 2.1 and 2.2 Isolates Circulating in China. Vet Microbiol (2019) 232:114–20. 10.1016/j.vetmic.2019.04.001 31030834

[4] Luo Y Ji S Lei JL Xiang GT Liu Y Gao Y. Efficacy Evaluation of the C-Strain-Based Vaccines Against the Subgenotype 2.1d Classical Swine Fever Virus Emerging in China. Vet Microbiol (2017) 201:154–61. 10.1016/j.vetmic.2017.01.012 28284603

[5] Coronado L Bohórquez JA Muñoz-González S Perez LJ Rosell R Fonseca O. Investigation of Chronic and Persistent Classical Swine Fever Infections Under Field Conditions and Their Impact on Vaccine Efficacy. BMC Vet Res (2019) 15:247. 10.1186/s12917-019-1982-x 31307464 PMC6632193

[6] Luo Y Li S Sun Y Qiu HJ. Classical Swine Fever in China: A Minireview. Vet Microbiol (2014) 172:1–6. 10.1016/j.vetmic.2014.04.004 24793098

[7] Tarradas J Argilaguet JM Rosell R Nofrarías M Crisci E Córdoba L. Interferon-Gamma Induction Correlates With Protection by DNA Vaccine Expressing E2 Glycoprotein Against Classical Swine Fever Virus Infection in Domestic Pigs. Vet Microbiol (2010) 142:51–8. 10.1016/j.vetmic.2009.09.043 19896784

[8] Meyer D Fritsche S Luo Y Engemann C Blome S Beyerbach M. The Double-Antigen ELISA Concept for Early Detection of E(rns)-Specific Classical Swine Fever Virus Antibodies and Application as an Accompanying Test for Differentiation of Infected From Marker Vaccinated Animals. Transbound Emerg Dis (2017) 64:2013–22. 10.1111/tbed.12611 28158921

[9] Gavrilov BK Rogers K Fernandez-Sainz IJ Holinka LG Borca MV Risatti GR. Effects of Glycosylation on Antigenicity and Immunogenicity of Classical Swine Fever Virus Envelope Proteins. Virology (2011) 420:135–45. 10.1016/j.virol.2011.08.025 21968199

[10] Lin GJ Deng MC Chen ZW Liu TY Wu CW Cheng CY. Yeast Expressed Classical Swine Fever E2 Subunit Vaccine Candidate Provides Complete Protection Against Lethal Challenge Infection and Prevents Horizontal Virus Transmission. Vaccine (2012) 30:2336–41. 10.1016/j.vaccine.2012.01.051 22300723

[11] Huang YL Deng MC Wang FI Huang CC Chang CY. The Challenges of Classical Swine Fever Control: Modified Live and E2 Subunit Vaccines. Virus Res (2014) 179:1–11. 10.1016/j.virusres.2013.10.025 24211665

[12] Skwarczynski M Toth I. Recent Advances in Peptide-Based Subunit Nanovaccines. Nanomed (Lond) (2014) 9:2657–69. 10.2217/nnm.14.187 25529569

[13] Karch CP Burkhard P. Vaccine Technologies: From Whole Organisms to Rationally Designed Protein Assemblies. Biochem Pharmacol (2016) 120:1–14. 10.1016/j.bcp.2016.05.001 27157411 PMC5079805

[14] Yang F Mariz FC Zhao X Spagnoli G Ottonello S Müller M. Broad Neutralization Responses Against Oncogenic Human Papillomaviruses Induced by a Minor Capsid L2 Polytope Genetically Incorporated Into Bacterial Ferritin Nanoparticles. Front Immunol (2020) 11:606569. 10.3389/fimmu.2020.606569 33343580 PMC7746619

[15] Negahdaripour M Golkar N Hajighahramani N Kianpour S Nezafat N Ghasemi Y. Harnessing Self-Assembled Peptide Nanoparticles in Epitope Vaccine Design. Biotechnol Adv (2017) 35:575–96. 10.1016/j.biotechadv.2017.05.002 PMC7127164 28522213

[16] Mohsen MO Zha L Cabral-Miranda G Bachmann MF. Major Findings and Recent Advances in Virus-Like Particle (VLP)-Based Vaccines. Semin Immunol (2017) 34:123–32. 10.1016/j.smim.2017.08.014 28887001

[17] He D Marles-Wright J. Ferritin Family Proteins and Their Use in Bionanotechnology. N Biotechnol (2015) 32:651–7. 10.1016/j.nbt.2014.12.006 PMC4571993 25573765

[18] Han JA Kang YJ Shin C Ra JS Shin HH Hong SY. Ferritin Protein Cage Nanoparticles as Versatile Antigen Delivery Nanoplatforms for Dendritic Cell (DC)-Based Vaccine Development. Nanomedicine-UK (2014) 10:561–9. 10.1016/j.nano.2013.11.003 24262997

[19] Zhu Y Jiang J Said-Sadier N Boxx G Champion C Tetlow A. Activation of the NLRP3 Inflammasome by Vault Nanoparticles Expressing a Chlamydial Epitope. Vaccine (2015) 33:298–306. 10.1016/j.vaccine.2014.11.028 25448112 PMC4272900

[20] Rome LH Kickhoefer VA. Development of the Vault Particle as a Platform Technology. ACS NANO (2013) 7:889–902. 10.1021/nn3052082 23267674

[21] Bennett KM Gorham RJ Gusti V Trinh L Morikis D Lo DD. Hybrid Flagellin as a T Cell Independent Vaccine Scaffold. BMC Biotechnol (2015) 15:71. 10.1186/s12896-015-0194-0 26265529 PMC4534063

[22] Giessen TW. Encapsulins: Microbial Nanocompartments With Applications in Biomedicine, Nanobiotechnology and Materials Science. Curr Opin Chem Biol (2016) 34:1–10. 10.1016/j.cbpa.2016.05.013 27232770

[23] Graham BS Gilman M McLellan JS. Structure-Based Vaccine Antigen Design. Annu Rev Med (2019) 70:91–104. 10.1146/annurev-med-121217-094234 30691364 PMC6936610

[24] Leneghan DB Miura K Taylor IJ Li Y Jin J Brune KD. Nanoassembly Routes Stimulate Conflicting Antibody Quantity and Quality for Transmission-Blocking Malaria Vaccines. Sci Rep (2017) 7:3811. 10.1038/s41598-017-03798-3 28630474 PMC5476561

[25] Bruun T Andersson AC Draper SJ Howarth M. Engineering a Rugged Nanoscaffold To Enhance Plug-And-Display Vaccination. ACS NAno (2018) 12:8855–66. 10.1021/acsnano.8b02805 PMC6158681 30028591

[26] Li Z Cui K Wang H Liu F Huang K Duan Z. A Milk-Based Self-Assemble Rotavirus VP6-Ferritin Nanoparticle Vaccine Elicited Protection Against the Viral Infection. J Nanobiotechno (2019) 17:13. 10.1186/s12951-019-0446-6 PMC6341625 30670042

[27] Gregson AL Oliveira G Othoro C Calvo-Calle JM Thorton GB Nardin E. Phase I Trial of an Alhydrogel Adjuvanted Hepatitis B Core Virus-Like Particle Containing Epitopes of Plasmodium Falciparum Circumsporozoite Protein. PloS One (2008) 3:e1556. 10.1371/journal.pone.0001556 18253503 PMC2216688

[28] Dhakal S Renukaradhya GJ. Nanoparticle-Based Vaccine Development and Evaluation Against Viral Infections in Pigs. Vet Res (2019) 50:90. 10.1186/s13567-019-0712-5 31694705 PMC6833244

[29] Yang L Li W Kirberger M Liao W Ren J. Design of Nanomaterial Based Systems for Novel Vaccine Development. Biomater Sci (2016) 4:785–802. 10.1039/C5BM00507H 26891972

[30] Salvador A Igartua M Hernández RM Pedraz JL. An Overview on the Field of Micro- and Nanotechnologies for Synthetic Peptide-Based Vaccines. J Drug Deliv (2011) 2011:181646. 10.1155/2011/181646 21773041 PMC3134826

[31] Rehm B. Bioengineering Towards Self-Assembly of Particulate Vaccines. Curr Opin Biotechnol (2017) 48:42–53. 10.1016/j.copbio.2017.03.018 28365472

[32] De Temmerman ML Rejman J Demeester J Irvine DJ Gander B De Smedt SC. Particulate Vaccines: On the Quest for Optimal Delivery and Immune Response. Drug Discov Today (2011) 16:569–82. 10.1016/j.drudis.2011.04.006 21570475

[33] Ambühl PM Tissot AC Fulurija A Maurer P Nussberger J Sabat R. A Vaccine for Hypertension Based on Virus-Like Particles: Preclinical Efficacy and Phase I Safety and Immunogenicity. J Hypertens (2007) 25:63–72. 10.1097/HJH.0b013e32800ff5d6 17143175

[34] Harding CV Song R. Phagocytic Processing of Exogenous Particulate Antigens by Macrophages for Presentation by Class I MHC Molecules. J Immunol (1994) 153:4925–33. 10.3389/fimmu.2018.02473 7963555

[35] López-Sagaseta J Malito E Rappuoli R Bottomley MJ. Self-Assembling Protein Nanoparticles in the Design of Vaccines. Comput Struct Biotechnol J (2016) 14:58–68. 10.1016/j.csbj.2015.11.001 26862374 PMC4706605

[36] Ding P Zhang T Li Y Teng M Sun Y Liu X. Nanoparticle Orientationally Displayed Antigen Epitopes Improve Neutralizing Antibody Level in a Model of Porcine Circovirus Type 2. Int J Nanomed (2017) 12:5239–54. 10.2147/IJN.S140789 PMC5533572 28769561

[37] Kanekiyo M Wei CJ Yassine HM McTamney PM Boyington JC Whittle JR. Self-Assembling Influenza Nanoparticle Vaccines Elicit Broadly Neutralizing H1N1 Antibodies. Nature (2013) 499:102–6. 10.1038/nature12202 PMC8312026 23698367

[38] Hsia Y Bale JB Gonen S Shi D Sheffler W Fong KK. Design of a Hyperstable 60-Subunit Protein Dodecahedron. [corrected]. Nature (2016) 535:136–9. 10.1038/nature18010 PMC4945409 27309817

[39] Xu H Wang Y Han G Fang W He F. Identification of E2 With Improved Secretion and Immunogenicity Against CSFV in Piglets. BMC Microbiol (2020) 20:26. 10.1186/s12866-020-1713-2 32019519 PMC7001342

[40] Zhang L Ren J Shi P Lu D Zhao C Su Y. The Immunological Regulation Roles of Porcine β-1, 4(B4GALT5) in PRRSV Infection. Front Cell Infect Microbiol (2018) 8:48. 10.3389/fcimb.2018.00048 29546034 PMC5837993

[41] OIE. Biosafety And Biosecurity Standard For Managing Biological Risk In The Veterinary Laboratory And Animal Facilities (2018). Available at: http://www.oie.int/fileadmin/Home/eng/Health_standards/tahm/1.01.04_BIOSAFETY_BIOSECURITY.pdf.

[42] Mittelholzer C Moser C Tratschin JD Hofmann MA. Analysis of Classical Swine Fever Virus Replication Kinetics Allows Differentiation of Highly Virulent From Avirulent Strains. Vet Microbiol (2000) 74:293–308. 10.1016/S0378-1135(00)00195-4 10831853

[43] Liu Z Liu Y Zhang Y Yang Y Ren J Zhang X. Surface Displaying of Swine IgG1 Fc Enhances Baculovirus-Vectored Vaccine Efficacy by Facilitating Viral Complement Escape and Mammalian Cell Transduction. Vet Res (2017) 48:29. 10.1186/s13567-017-0434-5 28499403 PMC5429525

[44] Pizza M Scarlato V Masignani V Giuliani MM Aricò B Comanducci M. Identification of Vaccine Candidates Against Serogroup B Meningococcus by Whole-Genome Sequencing. Science (2000) 287:1816–20. 10.1126/science.287.5459.1816 10710308

[45] Bachmann MF Jennings GT. Vaccine Delivery: A Matter of Size, Geometry, Kinetics and Molecular Patterns. Nat Rev Immunol (2010) 10:787–96. 10.1038/nri2868 20948547

[46] Chen G Bai Y Li Z Wang F Fan X Zhou X. Bacterial Extracellular Vesicle-Coated Multi-Antigenic Nanovaccines Protect Against Drug-Resistant Staphylococcus Aureus Infection by Modulating Antigen Processing and Presentation Pathways. THERANOSTICS (2020) 10:7131–49. 10.7150/thno.44564 PMC7330855 32641983

[47] Xia SL Lei JL Du M Wang Y Cong X Xiang GT. Enhanced Protective Immunity of the Chimeric Vector-Based Vaccine Radv-SFV-E2 Against Classical Swine Fever in Pigs by a Salmonella Bacterial Ghost Adjuvant. Vet Res (2016) 47:64. 10.1186/s13567-016-0346-9 27301745 PMC4908766

[48] Gao F Jiang Y Li G Li L Zhang Y Yu L. Evaluation of Immune Efficacy of Recombinant PRRSV Vectored Vaccine rPRRSV-E2 in Piglets With Maternal Derived Antibodies. Vet Microbiol (2020) 248:108833. 10.1016/j.vetmic.2020.108833 32891948

[49] Sun Y Li HY Tian DY Han QY Zhang X Li N. A Novel Alphavirus Replicon-Vectored Vaccine Delivered by Adenovirus Induces Sterile Immunity Against Classical Swine Fever. Vaccine (2011) 29:8364–72. 10.1016/j.vaccine.2011.08.085 21888938

[50] Pelliccia M Andreozzi P Paulose J D’Alicarnasso M Cagno V Donalisio M. Additives for Vaccine Storage to Improve Thermal Stability of Adenoviruses From Hours to Months. Nat Commun (2016) 7:13520. 10.1038/ncomms13520 27901019 PMC5141364

[51] Zou J Xie X Luo H Shan C Muruato AE Weaver SC. A Single-Dose Plasmid-Launched Live-Attenuated Zika Vaccine Induces Protective Immunity. EBIOMEDICINE (2018) 36:92–102. 10.1016/j.ebiom.2018.08.056 30201444 PMC6197676

[52] Lee BY Stalter RM Bacon KM Tai JH Bailey RR Zimmer SM. Cost-Effectiveness of Adjuvanted Versus Nonadjuvanted Influenza Vaccine in Adult Hemodialysis Patients. Am J Kidney Dis (2011) 57:724–32. 10.1053/j.ajkd.2010.12.016 PMC3085888 21396760

[53] Lin SY Chung YC Chiu HY Chi WK Chiang BL Hu YC. Evaluation of the Stability of Enterovirus 71 Virus-Like Particle. J Biosci Bioeng (2014) 117:366–71. 10.1016/j.jbiosc.2013.08.015 24140131

[54] Wang N Zhang Y Lei X Yu W Zhan Y Wang D. Optimized Conditions for Preserving Stability and Integrity of Porcine Circovirus Type2 Virus-Like Particles During Long-Term Storage. J Virol Methods (2017) 243:146–50. 10.1016/j.jviromet.2017.01.021 28131868

[55] Cruz-Reséndiz A Zepeda-Cervantes J Sampieri A Bastián-Eugenio C Acero G Sánchez-Betancourt JI. A Self-Aggregating Peptide: Implications for the Development of Thermostable Vaccine Candidates. BMC Biotechnol (2020) 20:1. 10.1186/s12896-019-0592-9 31959159 PMC6971912

[56] Yang F Wang F Guo Y Zhou Q Wang Y Yin Y. Enhanced Capacity of Antigen Presentation of HBc-VLP-Pulsed RAW264.7 Cells Revealed by Proteomics Analysis. J Proteome Res (2008) 7:4898–903. 10.1021/pr800547v 18842007

[57] Lycke N. Recent Progress in Mucosal Vaccine Development: Potential and Limitations. Nat Rev Immunol (2012) 12:592–605. 10.1038/nri3251 22828912

[58] Ganges L Núñez JI Sobrino F Borrego B Fernández-Borges N Frías-Lepoureau MT. Recent Advances in the Development of Recombinant Vaccines Against Classical Swine Fever Virus: Cellular Responses Also Play a Role in Protection. Vet J (2008) 177:169–77. 10.1016/j.tvjl.2007.01.030 17804267

[59] Graham SP Haines FJ Johns HL Sosan O La Rocca SA Lamp B. Characterisation of Vaccine-Induced, Broadly Cross-Reactive IFN-γ Secreting T Cell Responses That Correlate With Rapid Protection Against Classical Swine Fever Virus. VACCINE (2012) 30:2742–8. 10.1016/j.vaccine.2012.02.029 22366027

[60] Chackerian B. Virus-Like Particles: Flexible Platforms for Vaccine Development. Expert Rev Vaccines (2007) 6:381–90. 10.1586/14760584.6.3.381 17542753

[61] Crisci E Bárcena J Montoya M. Virus-Like Particles: The New Frontier of Vaccines for Animal Viral Infections. Vet Immunol Immunopathol (2012) 148:211–25. 10.1016/j.vetimm.2012.04.026 PMC7112581 22705417

[62] Kardani K Bolhassani A Shahbazi S. Prime-Boost Vaccine Strategy Against Viral Infections: Mechanisms and Benefits. Vaccine (2016) 34:413–23. 10.1016/j.vaccine.2015.11.062 26691569

[63] Cimica V Galarza JM. Adjuvant Formulations for Virus-Like Particle (VLP) Based Vaccines. Clin Immunol (2017) 183:99–108. 10.1016/j.clim.2017.08.004 28780375 PMC5673579

[64] Jain NK Sahni N Kumru OS Joshi SB Volkin DB Russell MC. Formulation and Stabilization of Recombinant Protein Based Virus-Like Particle Vaccines. Adv Drug Deliv Rev (2015) 93:42–55. 10.1016/j.addr.2014.10.023 25451136

[65] Kameyama KI Nishi T Yamada M Masujin K Morioka K Kokuho T. Experimental Infection of Pigs With a Classical Swine Fever Virus Isolated in Japan for the First Time in 26 Years. J Vet Med Sci (2019) 81:1277–84. 10.1292/jvms.19-0133 PMC6785620 31292349

[66] Leforban Y Cariolet R. Characterization and Pathogenicity for Pigs of a Hog Cholera Virus Strain Isolated From Wild Boars. Ann Rech Vet (1992) 23:93–100. 1387299

[67] Xia SL Du M Lei JL Liu Y Wang Y Ji S. Piglets With Maternally Derived Antibodies From Sows Immunized With Radv-SFV-E2 Were Completely Protected Against Lethal CSFV Challenge. Vet Microbiol (2016) 190:38–42. 10.1016/j.vetmic.2016.05.007 27283854

[68] Zhang H Wen W Zhao Z Wang J Chen H Qian P. Enhanced Protective Immunity to CSFV E2 Subunit Vaccine by Using IFN-γ as Immunoadjuvant in Weaning Piglets. Vaccine (2018) 36:7353–60. 10.1016/j.vaccine.2018.10.030 30366801

[69] Luo Y Ji S Liu Y Lei JL Xia SL Wang Y. Isolation and Characterization of a Moderately Virulent Classical Swine Fever Virus Emerging in China. Transbound Emerg Dis (2017) 64:1848–57. 10.1111/tbed.12581 27658930

[70] Everett H Salguero FJ Graham SP Haines F Johns H Clifford D. Characterisation of Experimental Infections of Domestic Pigs With Genotype 2.1 and 3.3 Isolates of Classical Swine Fever Virus. Vet Microbiol (2010) 142:26–33. 10.1016/j.vetmic.2009.09.039 19875252

[71] Fernandez-Sainz I Ramanathan P O’Donnell V Diaz-San SF Velazquez-Salinas L Sturza DF. Treatment With Interferon-Alpha Delays Disease in Swine Infected With a Highly Virulent CSFV Strain. Virology (2015) 483:284–90. 10.1016/j.virol.2015.04.024 26004252

