# Supplementary material for: Self-Assembling Nanovaccine Enhances Protective Efficacy Against CSFV in Pigs
Source: Front Immunol. 2021 Jul 21;12:689187. doi: 10.3389/fimmu.2021.689187 (PMC8334734; doi:10.3389/fimmu.2021.689187)
Supplement: Supplementary file 1 [file DataSheet_1.docx]

**Supplementary Material (additional method)**

**Generation of recombinant baculoviruses**

DNA sequences coding for a novel signal peptide (SP) directed truncated CSFV E2, were synthesized according to insect codon usage. The resulting gene was subcloned into baculovirus expression vector pFastBac HTA (Invitrogen, Carlsbad,CA, USA) between *Ehe* I and *Xho* I to generate HTA-SP-E2. mi3 encoding sequence (GenBank AXF54357.1) was introduced into HTA-SP-E2, with a flexible linker between E2 and mi3 to facilitate proper folding. All the plasmids were constructed in standard methods and verified by DNA sequencing. Recombinant baculoviruses were subsequently obtained by Tn7 transposition, bacmid extraction and transfection according to the manufacturer’s instructions. Protein Sequences are presented as following (tags and linkers underlined, and proteins in respective colors).

**>>SP-E2**MIKVLRGQVVQGIIWLLLVTGAQGRLSCKEDYRYAISSTNEIGPLGAEGLTTTWREYSHGLQLDDGTVRAICTAGSFKVIALNVVSRRYLASLHKRALPTSVTFELLFDGTSPTIEEMGDDFGFGLCPFDSTPVVKGKYNTTLLNGSAFYLVCPIGWTGVIECTAVSPTTLRTEVVKTYKREKPFPHRVDCVTTIVEKEDLFYCKWGGNWTCVKGNPVTYIGGQVKQCRWCGFDFKEPDGLPHYPIGKCILANETGYRVVDSTDCNRDGVVISTEGEHECLIGNTTVKVHALDGRLGPMPCRPKEIVSSAGPVRKTSCTFNYTKTLRNKYYEPRDSYFQQYMLKGEYQYWFDLDVHHHHHHHHHHHH

**>>SP-E2-mi3**

MIKVLRGQVVQGIIWLLLVTGAQGRLSCKEDYRYAISSTNEIGPLGAEGLTTTWREYSHGLQLDDGTVRAICTAGSFKVIALNVVSRRYLASLHKRALPTSVTFELLFDGTSPTIEEMGDDFGFGLCPFDSTPVVKGKYNTTLLNGSAFYLVCPIGWTGVIECTAVSPTTLRTEVVKTYKREKPFPHRVDCVTTIVEKEDLFYCKWGGNWTCVKGNPVTYIGGQVKQCRWCGFDFKEPDGLPHYPIGKCILANETGYRVVDSTDCNRDGVVISTEGEHECLIGNTTVKVHALDGRLGPMPCRPKEIVSSAGPVRKTSCTFNYTKTLRNKYYEPRDSYFQQYMLKGEYQYWFDLDVGGSGGSGGSGGSMKMEELFKKHKIVAVLRANSVEEAKKKALAVFLGGVHLIEITFTVPDADTVIKELSFLKEMGAIIGAGTVTSVEQCRKAVESGAEFIVSPHLDEEISQFCKEKGVFYMPGVMTPTELVKAMKLGHTILKLFPGEVVGPQFVKAMKGPFPNVKFVPTGGVNLDNVCEWFKAGVLAVGVGSALVKGTPVEVAEKAKAFVEKIRGCTEHHHHHHHHHHHH
